# Isolation by Distance, Source-Sink Population Dynamics and Dispersal Facilitation by Trade Routes: Impact on Population Genetic Structure of a Stored Grain Pest

**DOI:** 10.1534/g3.118.200892

**Published:** 2019-02-26

**Authors:** Erick M. G. Cordeiro, James F. Campbell, Thomas Phillips, Eduard Akhunov

**Affiliations:** * Department of Entomology, Kansas State University, Manhattan, KS 66506; † USDA, Agricultural Research Service, Center for Grain and Animal Health Research, 1515 College Ave., Manhattan, KS 66502; ‡ Department of Plant Pathology, Kansas State University, Manhattan, KS 66502

**Keywords:** population genetics, GBS, dispersal facilitation, stored pest, *Rhyzopertha dominica*

## Abstract

Population genetic structure of agricultural pests can be impacted not only by geographic distance and the broader ecological and physical barriers but also by patterns related to where crops are produced and how they are moved after harvest. Stored-product pests, for instance, specialize in exploiting grains such as wheat and rice from on-farm storage through transportation to final processing at often geographically distant locations; therefore human-aided movement may impact their dispersal. Although stored product insects are associated with stored grain, they can also exploit resources in the surrounding environments so different ecological regions where the grain is grown and stored may also influence population structure. Here we used 1,156 SNP markers to investigate how geographic distance, ecological and agricultural variables can impact the genetic structure and gene flow of the stored food pest beetle *Rhyzopertha dominica*. We found a substantial degree of admixture between weakly structured populations in the US. Ecological regions were more important in explaining *R. dominica* population structure than crop type, suggesting insect movement between wheat and rice grain distribution channels. We have also found a significant correlation between the genetic and geographical distance (*i.e.*, isolation by distance). However, our modeling approach combining the ecological and management variables has highlighted the importance of the volume of grain received by a location in the dispersal dynamics of the pest. The first-generation migrant analysis offered additional supported to movement over great distances that are likely associated with grain movement. Our data suggest that a multitude of factors play small but significant parts in the movement dynamics of the pest. The beetles can take advantage of the source-sink dynamic of grain movement in the US, but also engage in a high rate of movement at the local scale. Understanding population structure for *R. dominica* will provide insights into the potential for local processes of adaptation and broader patterns of movement that will impact management programs and the potential for spread of resistance genes.

Population structure of agricultural pests is likely impacted not only by geographic distance and the broader ecological and physical barriers but also by patterns related to where crops are produced and how they are moved after harvest. For example, grain commodities such as wheat and rice are typically grown in specific geographic regions where proper agronomic and environmental conditions can be found for the plant to develop. After harvest, the grain is often transported and stored multiple times, often over considerable geographic distances. Unwantedly, there is a community of organisms, including a number of significant stored product insect pests that can exploit the sizeable artificial resource patches created by storage of grain after harvest and are effective at finding and infesting these patches. The movement and storage of grain as it moves from producers to consumers generates a complex network of sources and receivers of grain that has the potential to facilitate the movement of associated pests (Dias 1996; Nopsa *et al.* 2015).

Source-sink dynamic predictions are primarily based on the differences in patch quality and availability, with higher quality and more available resources found in the source locations and lower quality and less available supply in the sink locations (Dias 1996). In theory, individuals in a source population have a higher rate of population growth due to more opportunities to find suitable habitat where they can feed and reproduce, and the opposite is expected for sink populations (Diffendorfer 1998). Over time, the source-sink model predicts that the source habitat is a net exporter of individuals and the sink is a net importer, with the assumption that dispersal is constrained so that individuals cannot be free to disperse to any or all possible locations (Holt 1997; Dias 1996; Diffendorfer 1998). This concept of source-sink has been applied to the network of grain storage and transportation and its potential impact on pests and pathogens associated with the grain (Nopsa *et al.*, 2015), but the actual implications of grain production and transportation on pest populations has not been evaluated. Areas with large amounts of grain production and storage are likely to be sources of grain to areas that have limited grain production and storage (*i.e.*, sink locations). The human designed grain production system and its network of storage and movement can facilitate the long-range dispersal of some insects (Drury *et al.* 2009; Semeao *et al.* 2012; Nopsa *et al.* 2015). The impact of this network can be extended to local adaptation, allelic diversity, development of resistance to pesticides, and the range of geographic distribution of the pest insects. This network is also fundamentally different for wheat and rice crops. Wheat is generally produced over a larger area and transported much further distances before processing compared with rice, which tends to be stored and milled within the same local area. Since there is limited overlap in the production areas and transportation network, populations of insects associated with each of these grains may be less likely to overlap.

One of the major insect pests of stored grain throughout the world is *Rhyzopertha dominica*, also known as the lesser grain borer (Potter 1935). Grain such as wheat and rice typically become colonized by *R. dominica* after it is harvested and stored, with infestations coming either from remaining populations on-site or movement of beetles into the storage by their flight dispersal or transportation in grain and grain handling equipment. This species can also be found in natural areas feeding on fruit seeds, shrub, dry-wood, and timber (Potter 1935; Wright *et al.* 1990; Jia *et al.* 2008); however, the influence of natural habitats on dynamics in grain storage is not well understood (Mahroof and Phillips 2007; Ching’Oma 2006). This small beetle is a strong flier, and cross-infestation among storage sites or from natural areas into storage sites is possible (Edde *et al.* 2012; Ching’Oma 2006), but little is known about their long-range dispersal ability (Sinclair and Haddrell 1985; Dowdy and McGaughey 1994; Toews *et al.* 2006).

One can find *R. dominica* throughout the United States, and it is established in all major wheat and rice production areas, although less abundant and less severe in the northern parts of the continent due to its limited cold tolerance (Edde 2012). Due to the broad geographical distribution of grain production, *R. dominica* is present in many different ecoregions (*i.e.*, geographically defined regions that are ecologically and environmentally similar) within the United States, including diverse habitats such as prairies, plains, mountains, valley, highlands, hills, and lowlands (EPA 1997). While the environment within grain storages, where insects can potentially spend many generations, is very homogeneous across these ecoregions, leading to more homogeneity in populations, local population genotype and dispersal ability may still be impacted by the surrounding environment. Ecoregions may also confound the effects of geographic distance and selection due to the influence of local conditions.

Our primary objective in this study was to estimate the relative importance of natural features of the environment (ecoregions), elements of the crop production system (wheat *vs.* rice), geographical distance, and human-aided movement (rail transportation network) on *R. dominica* population structure within the United States. To accomplish that, we collected beetles from different wheat and rice areas within the same season and conducted a hierarchical approach to test for possible structure-causing factors (Johnson and Omland 2004; Wagenmakers and Farrell 2004). As the association between *R. dominica* and stored grain is clearly undesirable, our results may help in the design of better monitoring tools and management strategies.

## Materials and Methods

### Sampling

**Figure 1:**
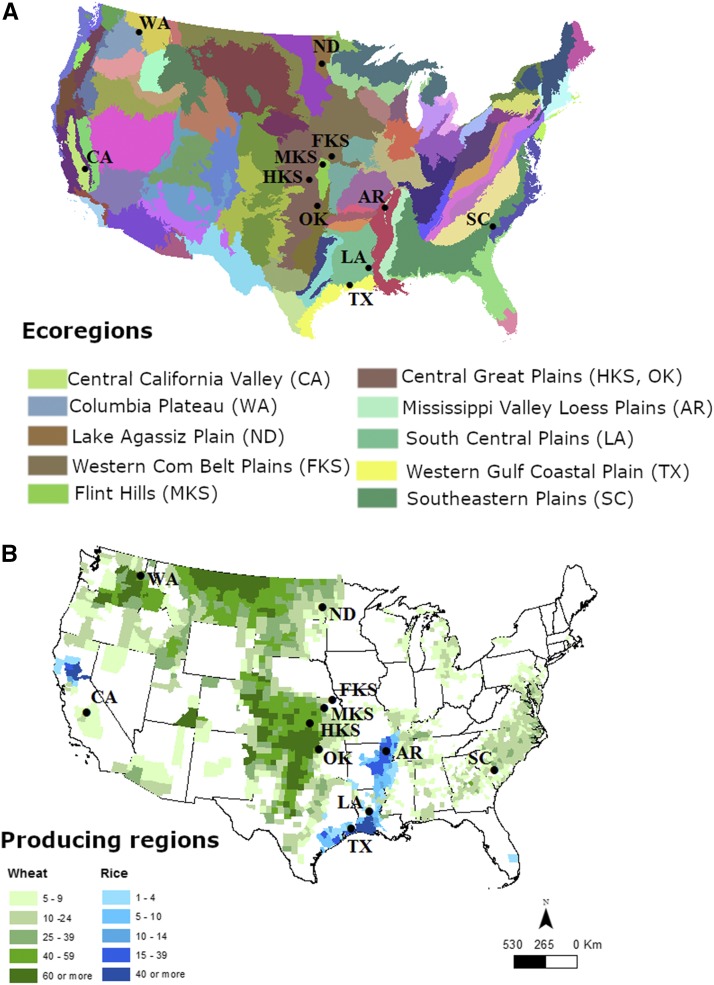
*Rhyzopertha dominica* sampling locations in the United States. (A) Ecoregions (legend refer only to ecoregions sampled according to EPA classification) and (available at https://www.epa.gov/eco-research/ecoregions-north-america) (B) wheat (green) or rice (blue) crop region. Map displaying ranges of all wheat and rice harvested for grain as a percent (%) of harvested cropland in 2012. Darker colors indicate a higher percent of the cropland acreage as all wheat and rice harvested for grain (source: NASS - USDA National Agricultural Statistic Service 2014). The locations LA and AR overlap wheat and rice areas. Source for the winter wheat and rice production are available at www.nass.usda.gov/Charts_and_Maps/Crops_County/.

We used *R. dominica* beetles collected at 11 distinct locations within wheat, rice, and wheat + rice production areas and within ten different ecoregions (EPA 1997) in the United States (Figure 1, Table 1). Ecoregions are geographical and ecological units that share similar features of environment, fauna, and flora (Bailey 2005). Sampling was done between July and November 2013, which is the period of the year when most flight activity is reported for this species (Edde *et al.* 2006; Toews *et al.* 2006). Six pheromone-baited delta traps (Scentry Biologicals Inc., Billing, MT) were deployed at each location targeting flying insects in the field near storage sites. The insect traps consisted of a cardboard sheet folded in a triangle shape with inner surfaces coated with sticky glue and containing a rubber septum lure impregnated with synthetic pheromone (Trece Inc., Adair OK) inside the trap. The traps were placed at least 10 m apart from each other and where possible close to grain storage sites such as grain bins, grain elevators, and warehouses. The pheromone lure within the traps can attract both male and female adults flying outdoors (Leos-Martinez *et al.* 1986; Toews *et al.* 2006). The traps were placed in the field for a week before being collected and for most locations shipped back to our lab where beetles were processed. For processing, the beetles were first carefully removed from the sticky glue inside the trap with forceps and transferred to a 15 ml tube containing 95% EtOH. A histological cleaning agent (Histo-Clear II, National Diagnostics; Atlanta, GA) was added to the tube and tube vortexed to remove residual glue from the beetles. The beetles were then transferred to a 1.5 ml tube with 95% EtOH and stored at -80° until DNA extraction. The number of beetles collected at each location varied due to both the number captured in traps and the integrity of the body after processing the sample. Due to variation in time of beetle capture and environmental conditions during the week the traps were deployed, some beetles became dehydrated which can decrease DNA extraction yield. Beetles that did not remain intact after processing were discarded to help reduce this effect.

**Table 1:** Sampling location information on ecoregion, crop type and number of individuals successfully genotyped (N_GEN_). R= rice, W= wheat

Ecological regions	Locations	Code	Lat	Long	Crop	N_GEN_
South Central Plains	Alexandria, LA	LA	31.2928	−92.4592	R|W	7
Western Gulf Coastal Plain	Beaumont, TX	TX	30.0649	−94.3325	R	29
Flint Hills	Manhattan, KS	MKS	39.1917	−96.5917	W	7
South central semiarid	Hudson, KS	HKS	38.0608	−97.9297	W	11
Western Corn Belt Plains	Fairview, KS	FKS	39.8464	−95.7337	W	5
Central California Valley	Parlier, CA	CA	36.6117	−119.526	W	42
Mississippi V. Loess Plains	Jonesboro, AR	AR	35.8281	−90.6942	R|W	30
South central semiarid	Stillwater, OK	OK	36.1157	−97.0586	W	11
Lake Agassiz Plain	Fargo, ND	ND	46.8772	−96.7894	W	43
Southeastern Plains	Orangeburg, SC	SC	33.4969	−80.8622	W	21
Columbia Plateau	Spokane, WA	WA	47.6589	−117.425	W	4

### DNA extraction and library preparation

Genomic DNA was extracted from individual *R. dominica* using the DNeasy kit (Qiagen, Valencia, CA) following the manufacturer recommended protocol. We eluted the DNA using distilled water and use a vacuum concentrator process (Speed Vac SC110, Savant) to concentrate samples to 50 μl DNA. RAD-sequencing (restriction site-associated DNA sequencing) libraries were prepared following Saintenac *et al.* (2013). Two libraries containing 80 individuals and one containing 96 individuals barcoded with unique nucleotide sequences were used. Complexity-reduced genomic libraries were prepared using the combination of two restriction endonucleases (RE), *PstI* (CTGCAG) and *MseI* (AATT), to create the genomic DNA fragments. After size selection targeting ∼300bp DNA fragment length, the size distribution of DNA fragments in the genomic libraries and the presence of contaminating adaptor peaks were tested on a Bioanalyzer (Agilent 2100), and real-time PCR was used to quantify libraries. Three libraries (pool of 80, 80, and 96 individuals) were diluted to 10 nM concentrations and sequenced on three lanes of Illumina HiSeq2000 (100 bp single-end read run) at the University of Kansas Genome Sequencing Core facility.

### Genotyping

*Rhyzopertha dominica* libraries were demultiplexed, separating individual beetles using the process_rad-tags script in STACKS v.1.44 (Catchen *et al.* 2013). The SNPs were identified on the 100 bp barcoded reads then filtered for overall quality (Ilut *et al.* 2014). A maximum of two nucleotide mismatches (M = 2) among reads with potentially variable sequences was the parameter used for the formation of RAD loci (Hohenlohe *et al.* 2011, Ilut *et al.* 2014; Benestan *et al.* 2015). The following filtering steps were taken using the population module of STACKS. We used RAD tags with a minimum stack depth (m) of 3 as this value has shown to perform well under other combinations of stack parameters (Hohenlohe *et al.* 2010, Hohenlohe *et al.* 2011, Rochette and Catchen 2017). Filtering was set to retain SNPs genotyped that were at least 85% of the individuals within locations and 100% of the among sampling locations (11 locations). After performing a series of preliminary tests combining different parameters for missing data allowance between and within the population (-p 11 and -r 0.85), the selected parameters were shown to be best in minimizing overall missing data and maximizing SNP retention for downstream analyses. We have also removed markers showing heterozygosity greater than 0.5 within samples to avoid potential paralogous loci (Hohenlohe *et al.* 2011; Benestan *et al.* 2015, Garcia-Elfring *et al.* 2017). The minor allele frequency (MAF) filtered out alleles with a frequency less than 5% (MAF< 0.05) to remove rare variants that have been shown to produce a biased estimate for population connectivity and population structure (Roesti *et al.* 2012; Linck and Battey 2017) (Table S2). Details of the number of SNPs kept after the filtering step were stored in variant call format (VCF), genepop, and structure files. Output files were converted when necessary into other file formats using PGDSPIDER 2.0 (Lischer and Excoffier 2012).

### Nucleotide diversity, population history, and migration inferences

Standard diversity indices for each location using SNP sets consisting of variant and invariant sites and genetic diversity analysis were calculated using the population module in STACKS. The number of variant sites, the number of polymorphic sites, effective number of alleles, the observed (H_o_) and expected heterozygosity (H_e_), inbreeding coefficient (F_IS_), and nucleotide diversity (π) across the genome were estimated for each sampled location. We have calculated allelic richness (A_R_) and private allelic richness (PA_R_) for each genetic cluster using rarefaction with standardized sample size of 8, equivalent to 4 diploid samples, with a tolerance of 15% missing data using ADZE 1.0 (Szpiech *et al.* 2008).

An assignment analysis was conducted, and the origin of each beetle was inferred by the calculation of the likelihood of a specific genotype to be found in a given population according to allele population frequencies using GENODIVE (Paetkau *et al.* 1995; Cornuet *et al.* 1999). Because the migration estimation methods based on *Nm* derived from F_ST_ can lack precision (Whitlock and McCauley 1999), the assignment method can be a viable alternative to infer migration using Monte Carlo resampling, in particular, first-generation migrants (Cornuet *et al.* 1999; Paetkau *et al.* 2004). The individual‐based assignment tests were performed on the multilocus genotype of each beetle using the assembly of individuals collected at the same place as a reference. We used the same set of markers as for the other analyses (1156 SNPs) that were shown to be within the optimum range for population assignment analysis (Benestan *et al.* 2015). We used a replacement rate of 0.005 (Paetkau *et al.* 2004) and Monte Carlo resampling for 10,000 permutations (Cornuet *et al.* 1999) and tested using the likelihood ratio threshold of all 11 locations at 0.002 alpha value.

#### Population clusters:

Population structure was tested using nested analysis of molecular variance (AMOVA) using 99,999 permutations with ARLEQUIN software in two hierarchical levels (Excoffier *et al.* 1992). The first hierarchical level tested for differences between crop group regions (rice or wheat) and the second level tested for differences between ecoregion within the crop group (Table 1). To visualize the level of gene flow between sampled locations and test number of clusters using genetic partitioning we used the software STRUCTURE v2.3.4 (Pritchard *et al.* 2000; Falush *et al.* 2003) using Bayesian clustering method with 1156 markers in STACKS. We used 1.5 × 10^−5^ burn-in iterations followed by 5 × 10^−6^ Markov Chain Monte Carlo (MCMC) steps. The analysis considered admixture model (*i.e.*, individuals may have mixed ancestry) and correlated allele frequencies (*i.e.*, frequencies in the different populations are likely to be similar). No prior information on the sampling location was included in the analysis. We have tested clusters from K = 1 to K = 12 with 10 replications. STRUCTURE output was analyzed using the program CLUMPP 1.1.2 (Jakobsson and Rosenberg 2007) and visualized using DISTRUCT (Rosenberg 2004). Most likely *K* values were selected with the Evanno method implemented in Structure Harvester (Earl and von Holdt 2012). We additionally performed a Principal Component Analysis (PCA) that is a non-model-based method to visualize possible factors that could explain grouping patterns (Zheng *et al.* 2012).

### F_ST_ and Isolation by Distance

Pair-wise F_ST_ analysis in Arlequin 3.5.2.2 was used to measure levels of genetic differentiation between two populations. We also assessed the population average pairwise matrix of Slatkin linearized F_ST_, population average pairwise differences, and population average pairwise matrix of M values (M = 2*Nm*). We used *RColorBrewer* and *igraph* packages to visualize the F_ST_ relations. We performed Mantel test with 10,000 permutations to correlate the genetic distances (F_ST_) matrix with the geographic distance matrix using the R libraries *ecodist* (Goslee and Urban 2007) and *ade4* (Dray and Dufour 2007).

### Model Selection

We used a model selection approach based on the Akaike’s Information Criterion (AIC, Wagenmakers and Farrell 2004) that allows several competing hypotheses to be simultaneously tested to find the single best model or a small set of very likely models (Johnson and Omland 2004). We selected and examined explanatory variables that can help us to predict the mean effective migrants exchanged by locations. Explanatory variables included geographical, bioclimatic, crop, and grain transport network variables. For geographic variables, we tested the relative effect of the average distance to the other locations (‘distance’) (Table S11). For the bioclimate variables, the PCA scores from the 19 WorldClim variables were used (Hijmans *et al.* 2005) (Table S12). For agriculture variables, we included crop production information such as the average wheat or rice acreage within a 50 km radius buffer from each sampling point. Buffer radius was selected based on the relative size of the zoned producing region and the ability of the insect to disperse (∼5 km) (Edde 2012). For instance, 50 km is large enough to encompass most regional rice-producing clusters but small enough to prevent significant overlap between landscapes. Landscapes were extracted from the wheat, and rice crop maps using ARGIS ESRI ArcMap v.10.0 and the average yield production for the state where the samples were collected (NASS - USDA National Agricultural Statistic Service 2014) (Table S13). For grain transportation variables, we tested how grain movement by railroads can potentially explain the observed variation in genetic parameters. We tested the volume of wheat received from other locations (‘received’), the volume of wheat shipped to other locations (‘shipped’), incoming and outgoing degree for each state, and betweenness centrality (‘centrality’). States with high incoming node degree are potentially important sinks (‘sink’) whereas those with high outgoing node degree are potentially important sources (‘source’). Betweenness centrality is the number of shortest paths going through a node in the rail network as a measurement of connectivity. A node with high betweenness centrality has a significant influence on the transfer of items through the system (Prater *et al.* 2013; Nopsa *et al.* 2015). Because available data on grain transportation is often summarized by state, we performed the analysis combining the three Kansas locations into a single value.

We standardized response and predictor variables (*i.e.*, converting to *z*-scores) prior to analysis so the beta could be interpreted as the standardized partial regression coefficient or beta weight (Abdi 2004). Beta weights can be compared and account for the relative contribution of each covariate present in the model. For each response variable, we tested the individual and additive effect of all covariates within a group of variables and then between groups of variables comparing the 200 top candidates. We selected models whose Corrected Akaike’s Information Criterion (AICc) values were less than two units away from the best model. The two-unit deviation criterion used to eliminate less plausible models from a smaller set of likely ones is somewhat arbitrary and therefore should not be used as an automatic cutoff. (Anderson 2007). In addition to the AICc ranked values, we have also chosen the best candidates according to model probabilities or Akaike weights (*w_i_*) (Johnson and Omland 2004). To separate the most import variables from less important ones, we used three criteria: a cutoff of 0.8 under the weight criterium, the frequency the variable appears in the top models, and beta weights (Burnham and Anderson 2003; Anderson 2008). We used *glmulti* (Calcagno and de Mazancourt 2010) package in R to perform model selection analyses using the exhaustive screening algorithm (Table S14).

### Data availability

A detailed supplementary document was prepared and made available at FigShare and GitHub at github.com/cordeiroemg. Tables and figures contain all the necessary information to reproduce the results presented here. Also, a fasta, genepop and VCF file containing all SNP information was made available. Supplemental material available at Figshare: https://doi.org/10.25387/g3.7242893.

## Results

### SNP discovery and data processing

The average number of sequence reads among the 3 libraries was 232.3 million (Library-1: 233.94, Library-2: 237.7, and Library-3: 225.19, Table S1) and the average percentage of quality-filtered reads (≥Q30) per library was 91.13% (Library-1: 92.3, Library-2: 90.8, and Library-3: 90.3), giving an average depth of coverage per individual over all SNPs of 35x (Figure S1). Average yield per library was 20.13 GB. Eighteen beetles (7% of the total) had insufficient mean coverage (<5x) and were removed from further analysis. After filtering steps, 45,7018 SNPs were retained before population module in STACKS when it was filtered down to 1,156 SNP markers.

### Nucleotide diversity, population history, and migration inferences

We had not found significant differences in nucleotide diversity among sampled locations when diversity parameters were calculated using only variant positions (Table 2) or using all positions (variant and fixed) (Table S4). Similar trend has been found for the rarefied allelic diversity and the rarefied private allelic richness indicating a lack of difference in genetic diversity among populations (Table 2). Considering all locations, the observed heterozygosity (H_0_) was 0.234 ± 0.137, the expected heterozygosity (H_E_) was 0.259 ± 0.136, and inbreeding coefficient (F_IS_) was 0.133 indicating fewer heterozygotes than expected (Supplementary material: Table S3 to S5).

**Table 2:** Genetic diversity statistics of *Rhyzopertha dominica* population from nine United States locations estimated from RADseq data (209 individuals and 1156 loci included) for only variant nucleotide positions; site polymorphisms, H_0_ observed heterozygosity, H_E_ expected heterozygosity, inbreeding coefficient (F_IS_) (mean ± 95% C.I.), nucleotide diversity (π) (mean ± 95% C.I.), rarefied allelic richness (A_R_) and rarefied private allelic richness (PA_R_) using a maximum standardized sample size of 8

Sample location	H_0_	H_E_	F_IS_	π	A_R_	PA_R_
Louisiana	0.227	0.225	0.041 ± 0.02	0.243 ± 0.01	1.640 ± 0.011	0.002 ± 0.001
Texas	0.219	0.234	0.062 ± 0.06	0.239 ± 0.01	1.616 ± 0.009	0.003 ± 0.001
Kansas	0.228	0.232	0.055 ± 0.01	0.250 ± 0.01	1.673 ± 0.008	0.004 ± 0.002
California	0.195	0.236	0.156 ± 0.08	0.239 ± 0.01	1.626 ± 0.009	0.003 ± 0.001
Arkansas	0.217	0.246	0.114 ± 0.07	0.251 ± 0.01	1.658 ± 0.008	0.003 ± 0.001
Oklahoma	0.219	0.238	0.091 ± 0.02	0.250 ± 0.01	1.660 ± 0.010	0.002 ± 0.001
North Dakota	0.228	0.251	0.090 ± 0.07	0.254 ± 0.01	1.667 ± 0.008	0.003 ± 0.001
South Carolina	0.235	0.245	0.054 ± 0.05	0.251 ± 0.01	1.655 ± 0.009	0.003 ± 0.001
Washington	0.226	0.208	0.028 ± 0.00	0.238 ± 0.01	1.589 ± 0.014	0.002 ± 0.001

The pair WA and TX showed the greatest pairwise nucleotide difference between populations (π_XY_ - (π_X_ + π_Y_)/2) followed by WA and LA and WA and SC (Table S6). The population pair with the smallest overall number of differences (*i.e.*, more similar to all other locations) was KS and OK. The population pair with the largest number of pairwise differences within the population (π_X_) (*i.e.*, more heterogeneous) was KS and WA whereas the population with the least amount of within population differences (*i.e.*, more homogenous) were CA and TX (Table S6). The estimated number of effective migrants *Nm* varied from 35.6 beetles between KS and ND to 5.1 beetles between CA and WA. The places that in average exchanged most migrants per generation were KS (24.5 beetles) and OK (23.2 beetles) whereas the places that exchanged the least were WA (5.9 beetles) and TX (9.1 beetles) (Table S9). The assignment analysis successfully assigned the majority of beetles to their correct collection location (78%), but there was considerable variation among locations (range: 0% LA to 98% CA). Beetles from SC, ND, TX, and CA were more often assigned to their correct locations, while LA and one of the KS populations (FKS) did not have any beetles assigned correctly. The high heterogeneity (high values for pairwise nucleotide difference within the population, π_X_) of central locations such as KS and OK can explain the low assignment rate found in those populations whereas TX, CA, SC were more homogenous. Two beetles were flagged as putative first-generation migrants: one beetle collected in Beaumont, TX, migrating from Jonesboro, AR, (L_h_/L_max_ (Threshold)= 173 (28.7)) and one collected in Orangeburg, SC, migrating from Parlier, CA (L_h_/L_max_ (Threshold)= 222.3 (65.5)). This result gives us strong evidence of a long-distance movement that is likely to be human-aided.

### Population structure and clustering

The Analysis of Molecular Variance (AMOVA) revealed a small but significant degree of genetic differentiation among ecoregion (Table 1) within the crop groups (F_SC_= 0.035, *P* = 0.001; Table 3), and between crops groups of wheat and rice (F_CT_= 0.006, *P* = 0.001; Table 3), using 1,156 SNP markers. The more substantial portion of the variance was allocated to the difference within groups (ecoregions) compared to differences between groups (crop type).

**Table 3:** F_ST_ variance within populations, F_SC_ variance among populations within crop groups, F_CT_ variance among crop groups (wheat *vs.* rice). The AR and LA location were assigned as rice location as that was the predominant crop

Source of variation	*d.f.*	SS	VC	%
Among group	1	269.487	0.42117	0.64
Among populations within groups	7	1117.794	2.26467	3.45
Within populations	409	25759.637	62.98200	95.91
Total	417	27146.919	65.66783	

**Figure 2:**
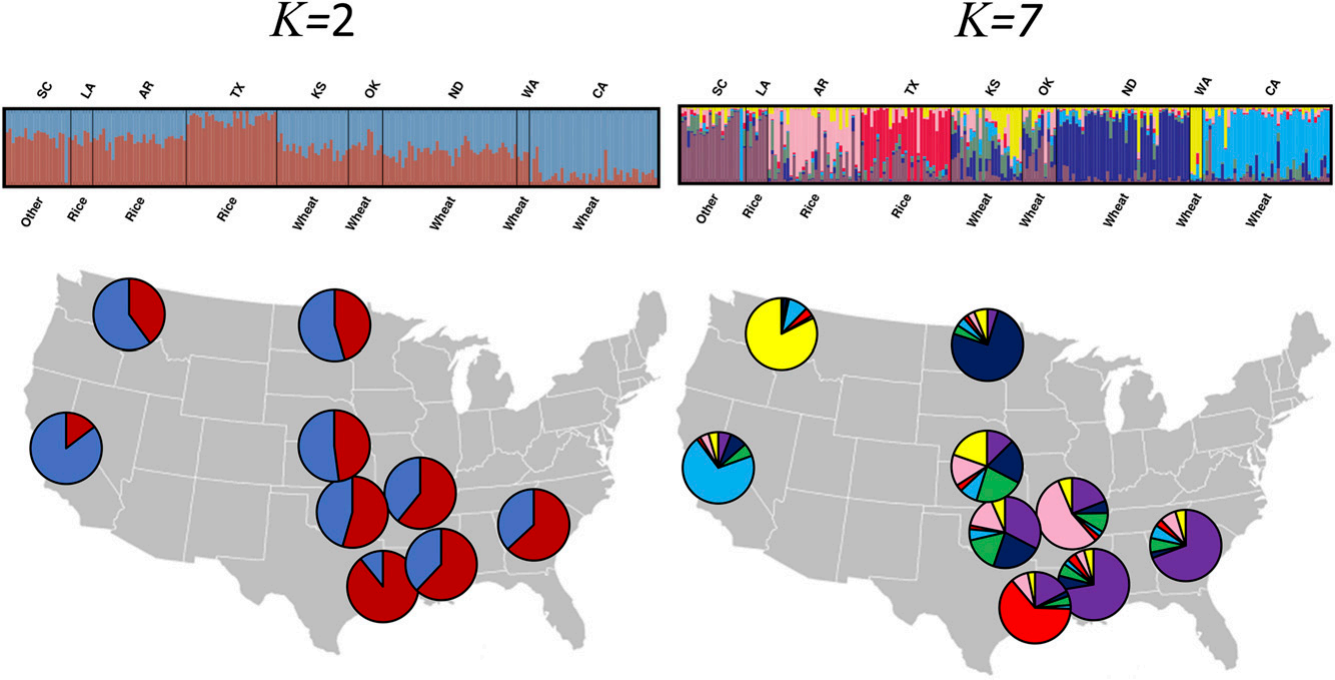
Structure plot of *R. dominica* across the United States. Vertical bars represent individuals whose genotype have been portioned into 2 and 7 clusters. Below each location is the predominate crop at the sampled region. Colors in pie charts represent the percentage of assignment (Q value) of each group).

Model-based and non-model-based cluster analyses agreed that optimum partition is two (Figure 2). The Evanno method estimated *K* = 2 as the most likely number of genetic clusters and the second most probably partition is *K* = 7 indicating a weaker but a possible level of substructure (Figure 2, Figure S2 and Table S10). Furthermore, evaluating other *K* values, it is possible to differentiate sampled locations as we increase *K* values (Figure S3). Genetic differentiation between wheat and rice regions was not apparent in the STRUCTURE analysis (Figure 2, Figure S3). The PCA analysis also reveals 2 clusters with a small intersection that could be linked to crop region (Figure S4) or the grain transport dynamic (Figure S5) even though the variance captured by the first principal components was relatively small.

### F_ST_ and Isolation by Distance

**Figure 3:**
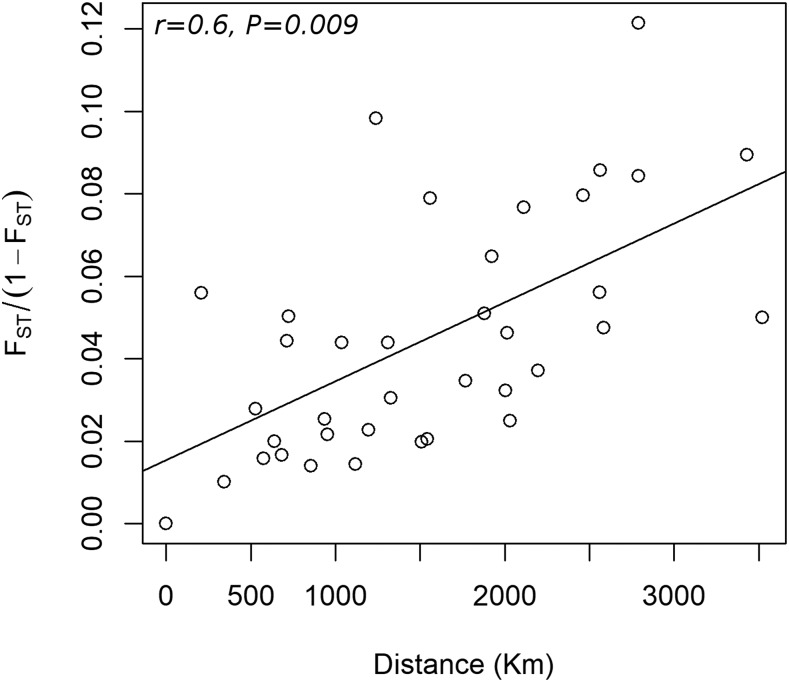
Isolation by distance (IBD) of *R. dominica* population. Analysis was based upon the correlation between genetic distance (F_ST_/1-F_ST_) and the geographic distance (km).

**Figure 4:**
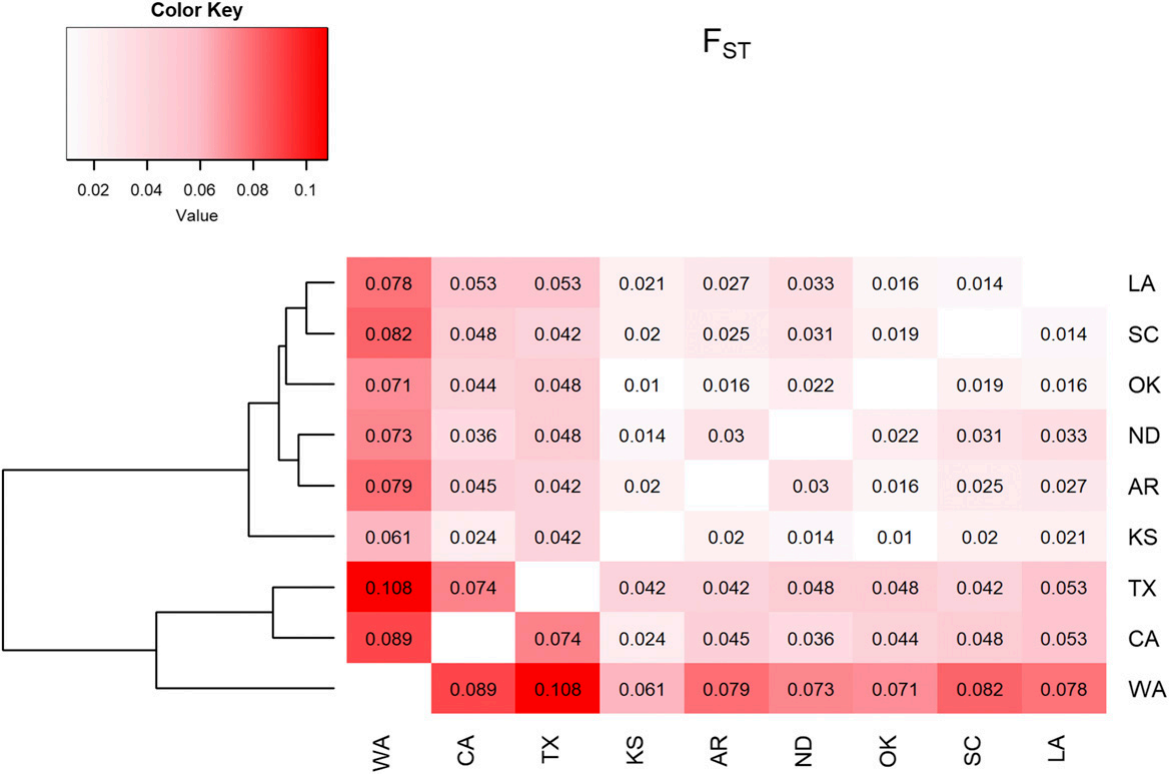
F_ST_ population dendogram and heatmap based on F_ST_ values Analysis compared a total of 11 *R. dominica* sampling locations. Darker color represents greater degree of differentiation. The three Kansas location were grouped together and for that reason considered a single location here.

**Figure 5:**
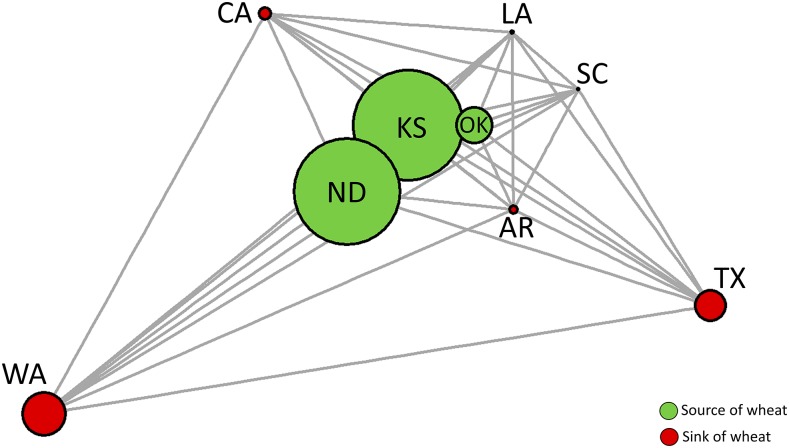
Network analysis of wheat producer and consumers. Network showing F_ST_ distance (links), wheat production (node size), and role as source (green) or sink (red) according to rail transport of wheat in the United States. Source locations are greater producers and tended be less isolated. Sink location are small producers and tented to be more isolated. KS, ND, OK are source populations of wheat whereas WA, CA, LA, SC, AR, TX are sink populations of wheat.

There was a significant association between genetic and geographic distances (Mantel’s r = 0.6, *P* = 0.009) considering all pairwise comparisons (Figure 3) and therefore evidence for isolation by distance (IBD). The level of genetic differentiation in pairwise comparisons varied 11-fold between the smallest and the highest degree of differentiation (F_ST_= 0.0101-0.108, Figure 4, Table S7, Table S8). The overall pattern also shows the formation of the two groups. The first group was formed by LA, SC, OK, ND, AR, and KS indicating that most locations the ‘Central Plains’ and ‘Coastal Plains’ are highly connected (Figure 4). The locations TX, CA formed the second group, and WA showing more differentiation in locations in the extremes of the range. By IBD showed in previous analyses, network analysis also reveals the importance of producers and consumers of grain in the population dynamics. Central nodes, which represent the largest wheat producers (OK, ND, and KS) and therefore sources of grain for other locations, are very close genetically to one another (Figure 5). Locations showing great genetic distance, on the other hand, tended to be associated with smaller producers and receivers of grain from source locations (Figure 5).

### Model selection

**Figure 6:**
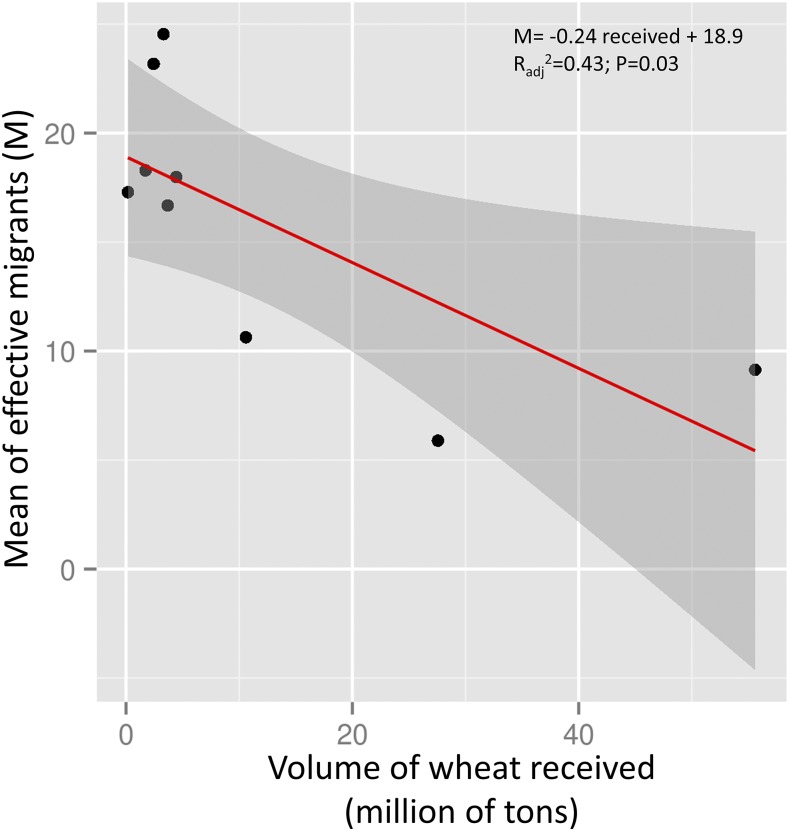
Probability of migrant exchanged according to wheat trade. Mean of effective migrant *Nm* as function of the volume of grain received (millions of tons) at each location.

A single model was selected to explain the average number of effective migrants (M) at each location (Figure S6). The top model elected volume of grain received and geographical distance as best predictors according to AIC_c_ criteria (top model AIC_c_ = 24.57 *vs.* null model AIC_c_= 30.48) (Table 4). The two predictors were consistent across the 100 models analyzed (Figure S7). Both variables were negatively associated with the average number of migrants, which means that locations closer to each other with the lower rates of grain receipts (*i.e.*, sources) tended to exchange more migrants with other locations. On the other hand, locations farther apart with a high rate of grain receipts (*i.e.*, sinks) tended to exchange fewer migrants with other locations. The volume of grain received at each location alone has a significant effect on the average number of effective migrants (M) and explains a significant portion of the variance (Figure 6).

**Table 4:** Global generalized linear model results (predictor variables included including intercept) modeling M (effective migrants)

M = Intercept + Distance + Receipts	Estimate	SE	t-value	Pr(>|t|)
Intercept	−2.18e-18	1.423e-01	0.000	1.0
Average geographic distance	−6.103e-01	1.532e-01	−3.984	0.00725***
Volume of grain received from other locations	−6.045e-01	1.532e-01	−3.946	0.00757***

* **significant at 0.001.

## Discussion

Here we used hundreds of markers to examine and extract genetic diversity information from local populations and found correlations with geographic distances, agriculture practices, and the movement of commodities across vast distances. For instance, agriculture-related variables such as the volume of grain received by a particular location helped to explain the degree of genetic isolation, and the number of effective migrants exchanged between populations. Here, we have not found statistically significant differences in genetic diversity among populations including nucleotide diversity, allele richness, and expected heterozygosity, which could be attributed to the high gene flow between areas. An interesting finding is that the most heterogeneous and better-connected populations are also located in places with the largest grain production in the United States. KS was the most heterogeneous location showing the highest value for pairwise nucleotide differences within population and lowest values of pairwise nucleotide differences between populations. More heterogenous areas tended to be large hubs of the grain transport network and highly connected to other areas. Our results support the notion that important production areas are support larger and heterogeneous populations (Nopsa *et al.* 2015).

### Heterozygosity deficiency

All populations showed lower values of observed heterozygosity than expected. Similar low heterozygosity has been described in the literature for other stored product insects (Demuth *et al.* 2007; Drury *et al.* 2009) and other beetle species (Brouat *et al.* 2003; Schrey *et al.* 2008). Heterozygosity deficiency may be associated with the way this species colonizes and disperses. Flying females outside of grain storage structures are often mated (Edde 2012, Ridley *et al.* 2016); thus, when infesting a large mass of grain such as a grain elevator or a grain bin, they may behave like a colonizing propagule. It is possible that the founding event that starts an infestation leads to a more inbred population (Wade and McCauley 1988). This effect can be substantial if the colonizing propagules are also coming from a single population or a small group of relatives (kin-structured colonization; Wade and McCauley 1988; Whitlock and McCauley 1990; Wade *et al.* 1994, Drury *et al.* 2009). Outside storage traps might reflect different patterns depending on the local dynamic at the specific sites where they were placed; *e.g.*, more significant differences among individuals flying toward the storage structure (colonization) and a higher relatedness for individuals flying away from a storage structure(dispersing).

Further research at the landscape scale level is needed to determine the genetic variability of *R. dominica* colonizers. For instance, it would be useful to determine if they tend to have higher levels of genetic similarity among themselves (originate from similar local sources), or if they will exhibit a higher level of genetic differentiation from one another (arise from different sources) as observed in *Tribolium* populations (Drury *et al.* 2009). The reunion of dispersing inbred colonizers from different locations can temporarily create substructure in the local population (Wade and McCauley 1988; Whitlock and McCauley 1990; Zhivotovsky 2015) that is likely to disappear within few generations of random mating (Berthier *et al.* 2006).

Heterozygous deficiency is not the only pattern that has been found in stored-product insects, other species have shown an excess of heterozygotes that was attributed to bottlenecks caused by fumigation or heat control tactics (Fields and White 2002; Semeao *et al.* 2012; Coelho-Bortolo *et al.* 2016; Blanc *et al.* 2006). Nonetheless, we must be careful interpreting and generalizing conclusions from inbreeding coefficients such as F_IS_ as such coefficients are more related to properties of the mating system within the population rather than evolutionary processes that lead to divergence among populations such as F_ST_ coefficients (Holsinger and Weir 2009).

### Population sub-division

*Rhyzopertha dominica* is a synanthropic insect that was likely first introduced into North America with grains brought by European settlers several hundred years ago, with many reintroductions likely to have occurred, and is now widely distributed across the US. However, this *trans*-continental distribution is not uniform throughout the range of this species, and we expected to encounter different degrees of isolation (Ellstrand and Elam 1993). Physical barriers such the Rocky Mountains and deserts, historical processes of human colonization, and characteristics of the insect dispersal behavior have an impact on gene flow and consequently on population structure (Lowe and Allendorf 2010). We have found a significant degree of isolation in populations from CA, WA, and TX, and because those locations also have the most substantial average distance to other places, isolation by distance (IBD) can be implied. Other studies on stored product insects have found a significant degree of structure but lack of IBD (Bas *et al.* 2000; Drury *et al.* 2009; Semeao *et al.* 2012; Coelho-Bortolo *et al.* 2016; Thangaraj *et al.* 2016), which led to the prevalent hypothesis that human-aided movement was a more critical factor than flight dispersal in defining population structure (Drury *et al.* 2009). Here we found that geographic distance can play a significant role in population structure even in stored-product insects with a high degree of predicted anthropogenic movement, at least at a large geographic scale. Distance can be considered an essential factor in determining the structure of stored product pests. When evaluating effective migrants *Nm*, the most significant number of migrants would be expected between KS and ND whereas KS was the place with the higher number of pairwise migrant exchange. When evaluating possible migrants in our samples, our assignment analysis tagged one insect from AR to TX and one from CA to SC as putative first-generation immigrants, which suggests both short and long longitudinal displacement between producing areas in the US. These findings strongly support the notion that beetles are dispersing with the grain using the transportation system (*i.e.*, train but potential not only) to engage in cross-continental travels.

Data presented here demonstrate that *R. dominica* populations experience a considerable amount of gene flow that was reflected in the high degree of population admixture illustrated in the structure plots. *Rhyzopertha dominica* populations seem to exhibit a combination of patterns, both continuous genetic differentiation and discrete population structure. The subtle levels of structure can give us clues of earlier demographic processes and ongoing evolutionary dynamics and therefore should be evaluated carefully. Keeping that in mind, we explored the population genetic structure using a wide range of techniques that indicated the influence of multiple and concurrent structuring factors. It appears that structuring factors might be to some extent correlated.

We presented two estimations for the number of clusters suggesting potential substructure. According to the Evanno method, *K* = 2 was the most likely number of groups, but there was also a small peak at *K* = 7. The smaller peak is much less important here, but it is nonetheless interesting to see that sampled location can still be identified despite the extremely high levels of gene flow. The PCA analysis, a non-model-based method, supported those two levels of structure even though it is not clear if crop type, source-sink dynamic, or another non-tested factor was responsible for the observed pattern. Scrutinizing *K* = 7, we confirm that KS and OK are indeed the most heterogeneous locations and CA, WA, and TX are the most homogenous. We can also spot the putative first-generation migrant in SC coming from CA. The F_ST_ values allowed the separation of the locations into two groups, one composed of relatively well-connected locations such as KS, ND, and OK and one composed by more structured locations (*i.e.*, higher F_ST_) such as WA, CA, and TX. Interestingly, TX is very close geographically to LA but seemed to be very distant genetically suggesting the importance of other processes and dynamics more complicated than just geographic distance.

### Crop *vs.* ecoregion

We found very little structure caused by crop region (*i.e.*, 0.63% of the total variance was explained by crop type). Given that rough rice tends not to be transported over very long distances prior to processing and is less likely to be cross-contaminated with wheat in transportation, we had predicted populations from rice-growing regions would be more isolated from wheat growing regions. However, our results are not conclusive regarding the impact of the crop because only the TX location could be considered an exclusive rice production region according to our classification (crop type within a 50 km radius). Our other rice-growing regions in AR and LA, while they can be considered rice regions predominantly, did also have some wheat grown within the established perimeter, making them mixed crop regions. That pattern of mixing areas was also apparent in the PCA plot. Texas presented a substantial degree of differentiation compared to a close location such as LA. These few cases of crop-related variation suggest that there may be a crop effect that was not detectable using our sample locations. The hypothesis that crop type could be a factor causing population structure has been investigated in the past in wheat and rice mills for *Tribolium* (Semeao *et al.* 2012), but in that study the variation within commodity type or region grouping was equal to or greater than that between groups, showing a weak relation at best. A finer scale sampling effort including more locations that are exclusively rice and wheat is necessary to definitively answer the question of whether crop type can be an essential factor structuring populations of *R. dominica*.

### Source-sink dynamics

Because distance has not been found as a major factor affecting population structure in stored-product insect populations in the past, grain transportation is often evoked as the best explanation for the lack of IBD (Bas *et al.* 2000; Blanc *et al.* 2006; Ryne and Bensch 2008). In the present work, we found a significant correlation between the geographic distance (km) and genetic distance (F_ST_/1-F_ST_) using the Mantel test, and a high rate of admixture (Figure 2). However, clear signs of other structuring factors related to agriculture activity and grain transportation suggest that they are potentially significant in impacting genetic parameters of the tested populations. Model selection allows us to test several competing hypotheses by weighting and establishing relationships between variables (Johnson and Omland 2004). We used geographic distance, crop information, bioclimatic, and transportation variables to assess the relative importance of each covariant for explaining genetic isolation and the number of migrants. The prediction was that larger geographic range over which grain is grown and the more grain shipped should be associated with greater number of migrants and less geographic isolation (source population, as in Semeao *et al.* 2012), and places that tend to receive more grain will have fewer migrants going out and are more isolated (sink population). Our prediction was supported because the covariant ‘volume of grain received’ was elected as most important considering all 100 models evaluated. This variable defines whether a location is a sink or a source (*i.e.*, low values of grain received are associated with source population, and high values of grain received are associated with sink population).

### Conclusions

Here we have shown how the geographic distance, features of the environment in which local populations are present, the agriculture management, as well as storage, and transportation of commodity are likely to affect different aspects of a stored grain insect pest population. This is the first time that population structure and IBD have been shown for a stored product insect. Inbreeding coefficients gave us insights on how populations infest and develop within grain storage sites; however, the observed heterozygosity deficiency needs further evaluation to determine if it is due to sample bias, such as timing before or after fumigation or being nearer to stored grain *vs.* natural habitat locations, or if it truly represents a pattern of substructure or kin-structure. Most heterogeneous and well-connected populations were in regions with the greatest wheat production. Given that after harvest wheat is typically stored locally and then transported through a series of successively larger and more distant storage locations, large production areas offer more opportunities for *R. dominica* to breed and these regions can potentially be a source of migrants to other less productive locations. In addition, dispersal by flight behavior and displacement from one location to another by human-aided means using trucks and railroads connecting populations can largely explain population differentiation patterns in *R. dominica* in the United States. This information also has a potential application to pest management and the management of insecticide resistance that needs further exploration.
